# Non-rigid MR-TRUS image registration for image-guided prostate biopsy using correlation ratio-based mutual information

**DOI:** 10.1186/s12938-016-0308-5

**Published:** 2017-01-10

**Authors:** Lun Gong, Haifeng Wang, Chengtao Peng, Yakang Dai, Min Ding, Yinghao Sun, Xiaodong Yang, Jian Zheng

**Affiliations:** 1 Suzhou Institute of Biomedical Engineering and Technology, Chinese Academy of Sciences, Suzhou, 215163 China; 2 University of Chinese Academy of Sciences, Beijing, 100049 China; 3 Department of Urology, Shanghai Changhai Hospital, Shanghai, 200433 China; 4 Department of Electronic Science and Technology, University of Science and Technology of China, Hefei, 230061 China; 5 School of Science, Nanjing University of Science and Technology, Nanjing, 210094 China

**Keywords:** Prostate, Needle biopsy, Non-rigid registration, CRMI, ISGD

## Abstract

**Background:**

To improve the accuracy of ultrasound-guided biopsy of the prostate, the non-rigid registration of magnetic resonance (MR) images onto transrectal ultrasound (TRUS) images has gained increasing attention. Mutual information (MI) is a widely used similarity criterion in MR-TRUS image registration. However, the use of MI has been challenged because of intensity distortion, noise and down-sampling. Hence, we need to improve the MI measure to get better registration effect.

**Methods:**

We present a novel two-dimensional non-rigid MR-TRUS registration algorithm that uses correlation ratio-based mutual information (CRMI) as the similarity criterion. CRMI includes a functional mapping of intensity values on the basis of a generalized version of intensity class correspondence. We also analytically acquire the derivative of CRMI with respect to deformation parameters. Furthermore, we propose an improved stochastic gradient descent (ISGD) optimization method based on the Metropolis acceptance criteria to improve the global optimization ability and decrease the registration time.

**Results:**

The performance of the proposed method is tested on synthetic images and 12 pairs of clinical prostate TRUS and MR images. By comparing label map registration frame (LMRF) and conditional mutual information (CMI), the proposed algorithm has a significant improvement in the average values of Hausdorff distance and target registration error. Although the average Dice Similarity coefficient is not significantly better than CMI, it still has a crucial increase over LMRF. The average computation time consumed by the proposed method is similar to LMRF, which is 16 times less than CMI.

**Conclusion:**

With more accurate matching performance and lower sensitivity to noise and down-sampling, the proposed algorithm of minimizing CRMI by ISGD is more robust and has the potential for use in aligning TRUS and MR images for needle biopsy.

## Background

Prostate cancer is among the most common diseases in males and exhibits a large and increasing morbidity in many countries. As the fifth leading cause of cancer death worldwide, prostate cancer affected approximately 1.1 million men worldwide in 2012 [1]. In China, for example, according to statistics, approximately 49,000 new cases of prostate cancer were reported, ranking ninth in terms of cancer incidence in men in 2011 [2]. Transrectal ultrasound-guided (TRUS) prostate biopsy is the most common means of diagnosing prostate cancer when an individual exhibits high blood levels of prostate-specific antigen (PSA). Although TRUS has many advantages, including real-time detection, low cost, and easy operation, its poor image quality and lack of clear contrast between malignant and normal tissue lead to false-negative rates of up to 30% for systematic sextant biopsies [3]. In contrast, magnetic resonance (MR) is the most sensitive imaging modality for observing anatomical structures and locating prostate tumor. Therefore, the registration of pre-operative MR images onto inter-operative TRUS images is of important clinical significance for improving biopsy accuracy.

Pre-operative and inter-operative prostate shape may suffer deformation due to extrusion of the ultrasonic probe, inflation of the anorectal coil inside the rectum during the MR scanning, and alterations in patient position [4]. To compensate for these movements, many non-rigid registration algorithms have been proposed over the past 20 years [5–8]. Mutual information (MI) is a widely used similarity criterion in multi-modal image registration and has been independently proposed by Collignon [9] and Viola [10]. Moradi [11] created a label map registration frame (LMRF) that aligned TRUS and MR images by using 3D Slicer, which first used the iterative closest point (ICP) method to rigidly align the outlines of the two images and then combined MI and B-splines to elastically register binary label maps from the two images obtained by manual contouring. Although LMRF aligned contours with high accuracy and could be conveniently implemented in 3D Slicer, it used only label maps to correct the local deformation and ignored pixel intensity, resulting in a high target registration error (TRE) of 3.6 ± 1.7 mm. Mitra [12] utilized directional quadrature filter pairs to convert TRUS and MR images into texture images and then used normalized mutual information (NMI) as a similarity criterion to register the texture images. The average TRE and mean Dice Similarity coefficient (DSC) were 2.64 ± 1.47 mm and 0.943 ± 0.039, but the computation time exceeded 797 s due to the use of 4 quadrature convolutions and the L-BFGS optimization method. However, several recent studies have shown that MI-based registration can be improved in certain cases. One improvement is to calculate MI over a set of overlapping image blocks to include spatial information. Loeckx [13] proposed the conditional mutual information (CMI), which considered the spatial location in the reference image of each joint intensity pair as the priori condition and calculated conditional entropies between the intensities given the spatial distribution. CMI-based registration resulted in a significant improvement in theoretical, phantom and clinical data compared with MI-based registration, but it was approximately 15 times slower than MI because it regarded the block label as the third channel of the joint histogram. Furthermore, MI assumes that all pixels in the overlapping area affect the calculations equally, but it is clear that different pixels contribute differently to the computation of MI [14]. Another kind of method assigns different weights to pixels using feature detection operators, e.g., the saliency measure [14] and the Harris corner detector [15]. But this method is often unable to extract effective features from TRUS images due to the low signal to noise ratio (SNR).

In this work, we design a mutual information-based method termed correlation ratio-based mutual information (CRMI) that includes the functional dependence of intensity values. MI only includes the correspondence of intensity classes to correct for the deformation of location but ignores the possible relationship between intensity values. We also deduce the analytical derivative of CRMI in detail. Furthermore, to improve the global searching ability and reduce the registration time, we introduce Metropolis acceptance criteria [16] to an stochastic gradient descent (SGD) optimizer, which increases both the random disturbance and the probability of escaping the local extremum.

## Methods

Let $V = \{ {\mathbf{x}} = (x,y)|0 \le x < S_{x} ,0 \le y < S_{y} \} \subseteq R^{2}$ denote the image domain. We use the MR image as a fixed image denoted by *F*(**x**) and the TRUS image as a moving image denoted by *M*(**x**). To simplify the derivation, we suppose that the intensities of the moving and fixed images have been normalized between zero and the number of histogram bins. The transformation that aligns *F* and *M* is represented by *T* = (*T*_*x*_, *T*_*y*_). The registration can be viewed as the problem of selecting the transformation that best minimizes a cost function

1 $$C = D(F({\mathbf{x}}),M({\mathbf{T}}({\mathbf{x}}))) + \mathop w\nolimits_{R} \cdot \mathop C\nolimits_{smooth} ({\mathbf{T}})$$

where *D* represents the similarity metric, *C*_*smooth*_ is the constraint of the grid that ensures its smoothness, as introduced by Rueckert [17], and *w*_*R*_ is the weight of the constraint used to balance the metric and the penalty term. *C*_*smooth*_ takes the following form in 2D:

2 $$\mathop C\nolimits_{smooth} ({\mathbf{T}}) = \frac{1}{N}\sum\limits_{{{\mathbf{x}} \in V}} {\left[ {\mathop {\left( {\frac{{\mathop \partial \nolimits^{2} {\mathbf{T}}}}{{\partial \mathop x\nolimits^{2} }}} \right)}\nolimits^{2} + \mathop {\left( {\frac{{\mathop \partial \nolimits^{2} {\mathbf{T}}}}{{\partial \mathop y\nolimits^{2} }}} \right)}\nolimits^{2} + 2 \times \mathop {\left( {\frac{{\mathop \partial \nolimits^{2} {\mathbf{T}}}}{\partial x\partial y}} \right)}\nolimits^{2} } \right]}$$

where **x** stands for the samples used to calculate the cost function and *N* is the total number of samples.

We choose a free-form deformation that is parameterized by the location of cubic B-spline nodes to simulate the transformation of the image. Given an *n*_*x*_ × *n*_*y*_ uniform grid of control points on the moving image with spacing *δ* and **u** as the location of the control points in the image plane. The deformation of a pixel at the coordinate (*x*, *y*) can be parameterized by **u** as follows:

3 $$T(x,y;{\mathbf{u}}) = \sum\limits_{l = 0}^{3} {\sum\limits_{z = 0}^{3} {\mathop B\nolimits_{l} (w)\mathop B\nolimits_{z} (v)} } \mathop u\nolimits_{{\mathop p\nolimits_{x} + l,\mathop p\nolimits_{y} + z}}$$

where $\mathop p\nolimits_{x} = \left\lfloor {x/\delta } \right\rfloor \,-\,{1,}\;\mathop p\nolimits_{y} = \left\lfloor {y/\delta } \right\rfloor \,-\,{1,}\;w\text{ = }x/\delta - \left\lfloor {x/\delta } \right\rfloor ,\;v = y/\delta \,-\, \left\lfloor {y/\delta } \right\rfloor$ and $\left\lfloor \cdot \right\rfloor$ is the truncating operation. *B* represents the cubic B-spline functions listed in Eq. (4). The multi-resolution Gaussian pyramid mentioned in [18] is applied to improve the searching efficiency.

4 $$\left\{ {\begin{array}{*{20}l} {\mathop B\nolimits_{0} (t) = \mathop {(1 - t)}\nolimits^{3} /6} \\ {\mathop B\nolimits_{1} (t) = (3\mathop t\nolimits^{3} - 6\mathop t\nolimits^{2} + 4)/6} \\ {\mathop B\nolimits_{2} (t) = ( - 3\mathop t\nolimits^{3} + 3\mathop t\nolimits^{2} + 3t + 1)/6} \\ {\mathop B\nolimits_{3} (t) = \mathop t\nolimits^{3} /6} \\ \end{array} } \right..$$

**Fig. 1:**
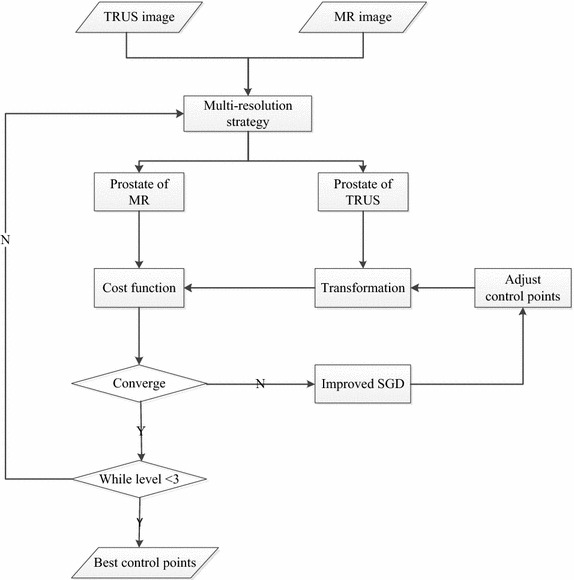
Schematic diagram of the proposed method

A schematic diagram of our method, which adopts a multi-resolution strategy, is shown in Fig. 1; each procedure will be illustrated in detail below.

### MI

MI measures the dispersion of the joint density based on the assumption of a correspondence of intensity classes between two images. It finds a balance between the maximization of the marginal entropies and the minimization of the joint entropy. Let *p*(*m*, *f*; **u**) be the joint probability density function of *M* and *F*, and let *p*(*m*; **u**) and *p*(*f*) be the marginal probability density functions of *M* and *F*, respectively. MI can be expressed as a function of control points **u** as follows:

5 $$MI(M,F;{\mathbf{u}}) = \sum\limits_{m,f} {p(m,f;{\mathbf{u}})} \mathop {\log }\nolimits_{2} \frac{{p(m,f;{\mathbf{u}})}}{{p(f)p(m;{\mathbf{u}})}} .$$

To derive the closed-form solution for the derivative of MI, we employ a second-order polynomial kernel designed by Xu [19] to estimate the probability density functions.

6 $$\mathop h\nolimits_{1} (t) = \left\{ {\begin{array}{*{20}l} { - 1.8\,\left| t \right|^{2} - 0.1\left| t \right| + 1,\;\;0 \le \left| t \right| <0.5} \\ {1.8\,\,\left| t \right|^{2} - 3.7\left| t \right| + 1.9,\;\;0.5 \le \,\left| t \right| \le 1} \\ {0, \qquad \qquad \qquad\qquad \;\, otherwise} \\ \end{array} } \right. .$$

**Fig. 2:**
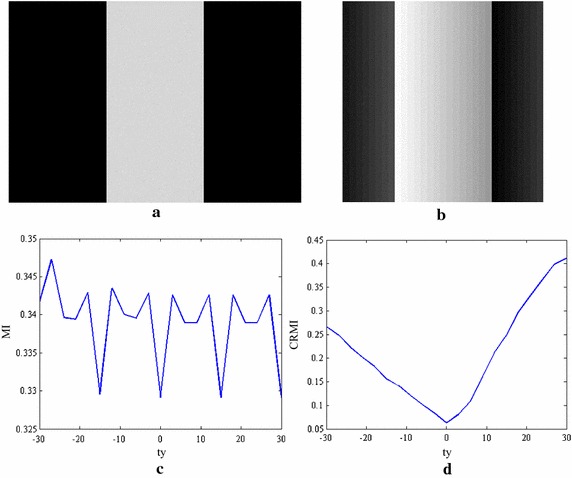
Registration experiments using synthetic data. **a** Binary image (450 by 310); **b** graded image of the stripe (310 by 310); **c** a plot of MI vs. horizontal translation; **d** a plot of CRMI vs. horizontal translation

However, MI only corrects the deformation of location and ignores the functional mapping of intensity values. MI-based registration may be inappropriate for the alignment of images with intensity distortion because the intensity bias will disperse the joint density. Figure 2 illustrates an example of registering two synthetic images, where Fig. 2b contains intensity distortion. Figure 2c displays the registration function of MI with respect to a horizontal shift of Fig. 2b, where the origin stands for an accurate alignment of the two images. It is clear that mutual information does not have an absolute minimum when the stripes completely overlap, possibly because the optimal alignment does not correspond to the assumption of intensity class correspondence.

On the other hand, MI requires enough samples to compute the probability density functions. This limits the application of MI in multi-resolution cases because the error between the estimation of the joint density and the real density will increase at low resolution. Furthermore, MI establishes a purely qualitative statistical relationship between the intensity classes, which means that the joint density is sensitive to noise.

### CR

For two random variables *X* and *Y*, the correlation ratio (CR) [20] is a quantitative description of the functional dependence between them, and the goal is to find the function *ϕ* ^*^ that best fits *Y* for all possible functions of *X*. Therefore, the problem is to find

7 $$\phi^{*} = \arg \;\mathop {\hbox{min} }\limits_{\phi } \left\| {Y - \phi (X)} \right\|_{2} .$$

Considering that the conditional expectation is the optimal approximator in the sense of the *L* ^2^ norm, the quality of fitting can be measured by CR as follows:

8 $$CR(Y,X) = 1 - \frac{Var[Y - E(Y|X)]}{Var[Y]}$$

where *Var*[*Y*] is the variance of *Y*, and $Var[Y - E(Y|X)]$ measures the part of *Y*that is functionally independent on *X* (see [20] for more details). CR varies between 0 (no functional dependence) and 1 (perfect functional relationship). In our method, the intensity values of the MR image are denoted as *X*, and the intensity values of the TRUS image are denoted as *Y*.

CR has been applied to register multi-modal images in [20–22]. Assuming a functional relationship between the intensities of float images and reference images is not an insurmountable obstacle for rigid or affine registration but is too restrictive to conform to the assumption for non-linear registration, especially when the image pairs have completely different presentations for the same anatomical structure. Therefore, CR is only applicable to rigid registration in most cases.

### CRMI

Although mapping all intensities between a float image and a reference image using one function may be over-constrained, it is reasonable to assume that the intensities of the aligned pixel pairs can be mapped by the same function. It seems feasible to combine the functional dependence of the intensity values with MI by utilizing the deformation parameters of MI-based registration as prior information to CR-based registration. However, this approach is not advisable because it might degrade the performance into the original CR-based method if the alignment obtained by MI is not reasonably accurate. Furthermore, the registration time is excessive due to the need to separately minimize MI and CR. Therefore, we extend the location alignment to include the functional mapping between the intensity pairs by multiplying MI with CR. The scatter-plot dispersion of intensity values is measured by combining the joint density of the intensity classes with the mapping relationship of the intensity values. We not only correct the location deformation, but also decrease the influence of intensity bias on the joint density.

As described in the literature [23], multiplication is preferred over addition because the addition of these terms requires normalization; therefore, we define a new similarity metric as follows:

9 $$CRMI\left( {M,F;{\mathbf{u}}} \right) = \left( {\mathop w\nolimits_{mi} - MI\left( {M,F;{\mathbf{u}}} \right)} \right) \cdot \left( {1 - CR\left( {M,F;{\mathbf{u}}} \right)} \right)$$

where *w*_*mi*_ is an empirical term, which ensures that CRMI is positive. In the new metric, *w*_*mi*_ is set to 1.5 and a linear kernel [24] that simplifies the derivation procedure is used to calculate CR as follow:

10 $$\begin{aligned} 1 - CR(M,F;{\mathbf{u}}) &= \frac{1}{{\mathop \sigma \nolimits^{2} }}\left( {\frac{1}{N}\sum\limits_{{\mathbf{x}} \in V} {\mathop M\nolimits^{2} ({\mathbf{T}}({\mathbf{x}};{\mathbf{u}}))} - \sum\limits_{f} {\mathop N\nolimits_{f} \mathop \mu \nolimits_{f}^{2} } } \right) \hfill \\ \mathop \mu \nolimits_{f} &= \frac{{\sum\nolimits_{{\mathbf{x}} \in V} {\mathop \lambda \nolimits_{{\mathbf{x}},f} M({\mathbf{T}}({\mathbf{x}};{\mathbf{u}}))} }}{{\mathop N\nolimits_{f} }},\quad \mathop N\nolimits_{f} = \sum\nolimits_{{\mathbf{x}} \in V} {\mathop \lambda \nolimits_{{\mathbf{x}},f} } \hfill \\ \end{aligned}$$

where *σ* ^2^ is the variance of the moving image, as for Eq. (11), and *λ*_**x**,*f*_ is the contribution of sample **x** to bin *f* as calculated by Eq. (12). *h*_2_(*t*) is the linear kernel as for Eq. (13), which ensures that each sample **x** in the MR assigns the weights to its two closest bins *f*_1_ − 1 and *f*_1_ ($\mathop f\nolimits_{1} = \left\lfloor {F({\mathbf{x}})} \right\rfloor \text{ + 1}$).

11 $$\mathop \sigma \nolimits^{2} = Var[M] = \frac{1}{N}\sum\limits_{{{\mathbf{x}} \in V}} {\mathop M\nolimits^{2} ({\mathbf{T}}({\mathbf{x}};{\mathbf{u}}))} - \bar{m}^{2} ,\quad \bar{m} = \frac{1}{N}\sum\limits_{{{\mathbf{x}} \in V}} {M({\mathbf{T}}({\mathbf{x}};{\mathbf{u}}))}$$

12 $$\mathop \lambda \nolimits_{{{\mathbf{x}},f}} = \frac{1}{N}\mathop h\nolimits_{2} (f - F({\mathbf{x}}))\sum\limits_{m = 0} {\mathop h\nolimits_{2} (m - M({\mathbf{T}}({\mathbf{x}};{\mathbf{u}})))} = \frac{1}{N}\mathop h\nolimits_{2} (f - F({\mathbf{x}}))$$

13 $$h_{2} (t) = \left\{ \begin{array}{ll} 1 - | t|,&\quad {\text{if}}\,0 \le | t| < 1 \\ 0, &\quad {\text{otherwise}}{\text{.}} \\ \end{array} \right.$$

Figure 2d shows the translational results of CRMI with respect to the horizontal shift. It can be observed that the registration function of CRMI has the only one global minimum that agrees with the correct transformation parameters. This finding demonstrates that CRMI is more reliable for measuring the scatter-plot dispersion of intensity values.

### Gradient

To take advantage of the gradient-based optimization algorithm, we should obtain the derivative of the cost function. The gradient of the cost function with respect to the control points **u** can be calculated as follows:

14 $$\frac{\partial C}{{\partial {\mathbf{u}}}} = \left( {1 - CR} \right)\frac{{\partial (\mathop w\nolimits_{mi} - MI)}}{{\partial {\mathbf{u}}}} + (\mathop w\nolimits_{mi} - MI)\frac{\partial (1 - CR)}{{\partial {\mathbf{u}}}} + \mathop w\nolimits_{reg} \frac{{\partial \mathop C\nolimits_{smooth} ({\mathbf{T}})}}{{\partial {\mathbf{u}}}}.$$

For an arbitrary control point *u*_*i*,*j*_, we derive the derivative of the three terms independently.

The derivative of MI was obtained in [18] as follows:

15 $$\frac{{\partial (\mathop w\nolimits_{mi} - MI)}}{{\partial \mathop u\nolimits_{i,j} }} = - \sum\limits_{m,f} {\mathop {\log }\nolimits_{2} } \left( {\frac{{p(m,f;{\mathbf{u}})}}{{p(m;{\mathbf{u}})}}} \right)\frac{{\partial p(m,f;{\mathbf{u}})}}{{\partial \mathop u\nolimits_{i,j} }}.$$

According to the chain rule, the last term in Eq. (15) can be calculated as follows:

16 $$\begin{aligned} \frac{{\partial p(m,f;{\mathbf{u}})}}{{\partial \mathop u\nolimits_{i,j} }} &= \frac{1}{N}\sum\limits_{{{\mathbf{x}} \in V}} {\left[ {\mathop h\nolimits_{1} (f - F({\mathbf{x}}))\frac{{d\mathop h\nolimits_{1} (\mathop \xi \nolimits_{{\mathbf{x}}} )}}{{d\mathop \xi \nolimits_{{\mathbf{x}}} }}|_{{\mathop \xi \nolimits_{{\mathbf{x}}} = m - M({\mathbf{T}}({\mathbf{x}};{\mathbf{u}}))}} } \right.} \\ & \quad \left. { \times \mathop {\left( {\frac{{ - \partial M({\mathbf{y}})}}{{\partial {\mathbf{y}}}}|_{{{\mathbf{y}} = {\mathbf{T}}({\mathbf{x}};{\mathbf{u}})}} } \right)}\nolimits^{T} \times \frac{{\partial {\mathbf{T}}({\mathbf{x}};{\mathbf{u}})}}{{\partial \mathop u\nolimits_{i,j} }}} \right]\end{aligned}$$

where $dh_{1} (\xi )/d\xi_{{\mathbf{x}}}$ is the first-order derivative of the kernel, $\partial M({\mathbf{y}})/\partial {\mathbf{y}}$ is the gradient of the moving image, and $\partial {\mathbf{T}}\left( {{\mathbf{x}};{\mathbf{u}}} \right)/\partial u_{i,j}$ is the derivative of the deformation field with respect to *u*_*i*,*j*_, the definition of which is given as follows:

17 $$\frac{{\partial {\mathbf{T}}({\mathbf{x}};{\mathbf{u}})}}{{\partial \mathop u\nolimits_{i,j} }} = \left\{ {\begin{array}{*{20}l} {\mathop B\nolimits_{r} (w)\mathop B\nolimits_{q} (v), \quad \left| {{\mathbf{x}} - \mathop u\nolimits_{i,j} } \right| \le 2\delta } \\ {0,\quad \qquad \quad \quad \, otherwise} \\ \end{array} } \right.$$

where *r* = *i* − *p*_*x*_, *q* = *j* − *p*_*y*_ and *p*_*x*_, *p*_*y*_, *w*, *v* are defined in Eq. (3).

Using the chain rule, the derivative of the second term in CR can be expressed as follows:

18 $$\frac{\partial (1 - CR)}{{\partial \mathop u\nolimits_{i,j} }} = \sum\limits_{{{\mathbf{x}} \in V}} {\left[ {\frac{\partial (1 - CR)}{{\partial \mathop \xi \nolimits_{{\mathbf{x}}} }}|_{{\mathop \xi \nolimits_{{\mathbf{x}}} = M({\mathbf{T}}({\mathbf{x}};{\mathbf{u}}))}} \times \mathop {\left( {\frac{{\partial M({\mathbf{y}})}}{{\partial {\mathbf{y}}}}|_{{{\mathbf{y}} = {\mathbf{T}}({\mathbf{x}};{\mathbf{u}})}} } \right)}\nolimits^{T} \times \frac{{\partial {\mathbf{T}}({\mathbf{x}};{\mathbf{u}})}}{{\partial \mathop u\nolimits_{i,j} }}} \right]}$$

where $\partial M({\mathbf{y}})/\partial {\mathbf{y}}$ and $\partial {\mathbf{T}}\left( {{\mathbf{x}};{\mathbf{u}}} \right)/\partial u_{i,j}$ are as defined in Eq. (16), and $\partial (1 - CR)/\partial \xi_{{\mathbf{x}}}$ is derived as follows:

19 $$\begin{aligned} \frac{\partial (1 - CR)}{{\partial \mathop \xi \nolimits_{{\mathbf{x}}} }}\big|_{{\mathop \xi \nolimits_{{\mathbf{x}}} = M({\mathbf{T}}({\mathbf{x}};{\mathbf{u}}))}} &= \frac{{ - \partial \mathop \sigma \nolimits^{2} /\partial \mathop \xi \nolimits_{{\mathbf{x}}} }}{{\mathop \sigma \nolimits^{4} }}\left( {\frac{1}{N}\sum\limits_{{{\mathbf{x}} \in V}} {\mathop \xi \nolimits_{{\mathbf{x}}}^{2} } - \sum\limits_{f} {\mathop N\nolimits_{f} \mathop \mu \nolimits_{f}^{2} } } \right) \\ & \quad + \frac{1}{{\mathop \sigma \nolimits^{2} }}\partial \frac{{\left( {\frac{1}{N}\sum\nolimits_{{{\mathbf{x}} \in V}} {\mathop \xi \nolimits_{{\mathbf{x}}}^{2} } - \sum\nolimits_{f} {\mathop N\nolimits_{f} \mathop \mu \nolimits_{f}^{2} } } \right)}}{{\partial \mathop \xi \nolimits_{{\mathbf{x}}} }} \end{aligned}$$

After calculating the derivatives and simplifying, we obtain

20 $$\begin{aligned} \left.\frac{\partial (1 - CR)}{{\partial \xi_{{\mathbf{x}}}}}\right|_{{\xi_{{\mathbf{x}}} = M({\mathbf{T}}({\mathbf{x}};{\mathbf{u}}))}} & = \frac{2}{{N \sigma^{2} }}\left( \xi_{{\mathbf{x}}} - N({\lambda }_{{{\mathbf{x}},f_{1} - 1}} \mu_{{f_{1} - 1}} + \lambda_{{{\mathbf{x}},f_{1} }}\mu_{{f_{1} }} ) - \frac{1}{{\sigma^{2} }}\left( {\xi_{{\mathbf{x}}} - \bar{m}} \right) \right. \\ & \quad \left. \left( {\frac{1}{N}\sum\limits_{{{\mathbf{x}} \in V}} {\xi_{{\mathbf{x}}}^{2} } - \sum\limits_{f} {N_{f} \mu_{f}^{2} } } \right) \right)\end{aligned}$$

where *f*_1_ and $\bar{m}$ are as defined in Eq. (11). The details of the derivation of Eq. (20) from Eq. (19) are given in Appendix 1. Combining Eq. (18) with Eq. (20), we can analytically derive the closed-form derivative of CR with respect to *u*_*i*,*j*_.

The derivative of the third formula *C*_*smooth*_ is given by Eq. (21), and the calculation of the right side of the equation is presented in Appendix 2.

21 $$\frac{{\partial \mathop C\nolimits_{smooth} ({\mathbf{T}})}}{{\partial \mathop u\nolimits_{i,j} }} = \frac{1}{N}\sum\limits_{{{\mathbf{x}} \in V}} {\left[ {2\frac{{\mathop \partial \nolimits^{2} {\mathbf{T}}}}{{\partial x^{2} }}\frac{{\partial (\mathop \partial \nolimits^{2} {\mathbf{T}}/\partial x^{2} )}}{{\partial \mathop u\nolimits_{i,j} }} + 2\frac{{\mathop \partial \nolimits^{2} {\mathbf{T}}}}{{\partial y^{2} }}\frac{{\partial (\mathop \partial \nolimits^{2} {\mathbf{T}}/\partial y^{2} )}}{{\partial \mathop u\nolimits_{i,j} }} + 4\frac{{\mathop \partial \nolimits^{2} {\mathbf{T}}}}{\partial x\partial y}\frac{{\partial (\partial^{2} {\mathbf{T}}/\partial x\partial y)}}{{\partial \mathop u\nolimits_{i,j} }}} \right]} .$$

### Optimization

A typical gradient-based optimization method using the set of parameters **u** can be expressed as follows:

22 $$\mathop {\mathbf{u}}\nolimits_{{{\mathbf{k + 1}}}} = \mathop {\mathbf{u}}\nolimits_{k} - \mathop a\nolimits_{k} \mathop {\mathbf{d}}\nolimits_{k} ,\quad k = 1,2, \ldots$$

where **d**_*k*_ is the search direction in iteration *k*, and *a*_*k*_ is the step size along the search direction. Klein [25], after comparing the performances of eight optimization methods, showed that SGD based on the Robbins-Monro algorithm was the best choice in most medical image registration applications. The main idea of SGD is to use an approximate gradient that is computed using a small, randomly picked subset of pixels to replace the real gradient calculated using all pixels, a method that is more efficient per iteration while not affecting the final accuracy [26]. In practice, SGD always exploits a choice such as that expressed in Eq. (23) to select the step size; thus, it is very difficult to determine the optimal values of the three parameters *a*, *A* and *α*.

23 $$\mathop a\nolimits_{k} = a(k) = \frac{a}{{\mathop {(k + A)}\nolimits^{\alpha } }}.$$

Klein [27] automatically calculated these parameters based on the distribution of the voxel displacements using the adaptive stochastic gradient descent method (ASGD); however, this method required computation of the Jacobin matrix of the deformation model with respect to the transformation parameters, which would be unacceptable if the number of transformation parameters was large. Qiao [28] proposed fast ASGD on the basis of ASGD to estimate the three parameters. This approach simplified the calculation of the bias and variance of the voxel displacements by using frequency statistics, a method that improved the efficiency at the cost of some precision. In addition, SGD requires a sufficient number of iterations to achieve convergence. The maximum number of iterations is also difficult to select. If the number is large, computation time will be increased; otherwise, convergence might not be achieved. Therefore, it is important to achieve the optimal efficiency and precision by setting up an appropriate stop condition.

In this section, we propose a new way to improve the performance of SGD, termed improved SGD (ISGD). The traditional SGD method can be viewed as a non-feedback system that does not depend on the exact value of the cost function. In our work, for each iteration, this value is fed back to the control loop to select a suitable step size, and its invariance within 25 consecutive iterations is used as the stop condition. Note that we utilize the discrete histogram to calculate the exact value of the cost function to save the computation time, but the registration accuracy is not affected because this value is only used for auxiliary judgment. To increase the robustness of ISGD with respect to the parameters used and to prevent falling into the local extremum, we introduce Metropolis acceptance criteria into the stochastic process as follows: If the current exact value is smaller than the previous value, the step size will be accepted; otherwise, the Boltzmann probability factor computed by Eq. (24) will be compared with a small random number distributed in the interval (0, 1). If the probability is larger than the random number, the current step size will be accepted. Otherwise, the current value is moving away from the optimal value, and the step size is decreased.

24 $$p = \exp \left( { - \frac{{C - \mathop V\nolimits_{b} }}{{\mathop T\nolimits_{0} }}} \right)$$

where *C* and *V*_*b*_ are the exact values of the cost function for the current and last iterations, respectively, and *T*_0_ is a constant.

Using the exact cost value to form a closed-loop system, ISGD can be supervised to find the optimal maximum number of iterations. To avoid immature convergence, we add a random disturbance on the basis of SGD by including Metropolis acceptance criteria. This procedure increases the probability that ISGD will jump out of the local extremum. ISGD will make it easier to choose the three parameters, because we only need to ensure that the initial step is large, and the procedure can always find a suitable step size after several attempts.

**Algorithm 1.**
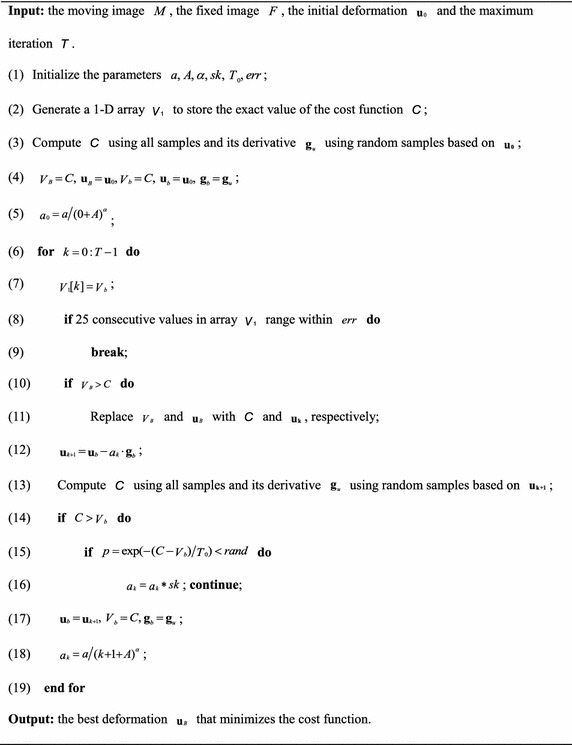
The pseudo code for the new optimization algorithm

## Experiments and results

In this section, we validate the effective performance of the proposed method using synthetic data and clinical prostate data. The proposed method is implemented in C++ and run on a PC equipped with an Intel Core i7 3.60 GHz CPU and 8 GB RAM.

### Synthetic data

**Fig. 3:**
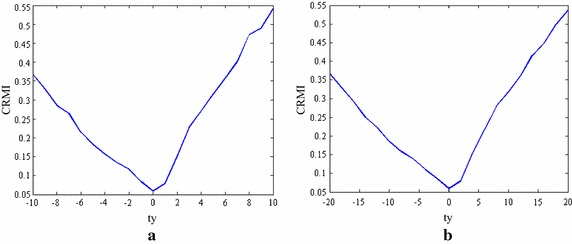
Registration functions for down-sampling. **a** Shows the functions of CRMI using the down-sample factor 4 × 4, and **b** shows the functions using the down-sample factor 2 × 2

**Fig. 4:**
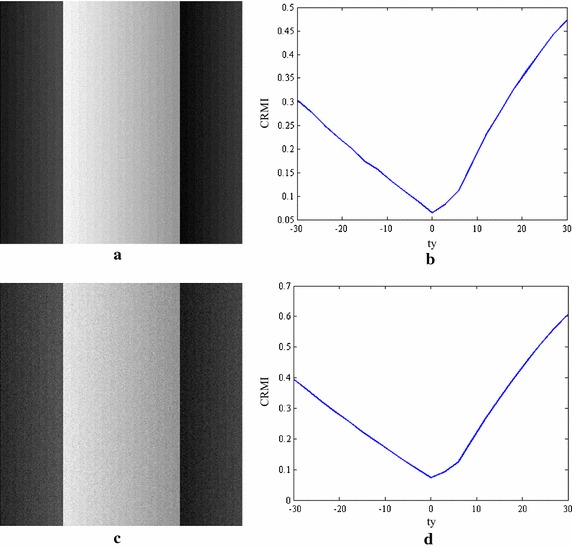
Noise image registration experiments. **a** shows the images with 10-dB noise, and **b** shows the corresponding registration function, **c** shows the image with 20-dB noise, and **d** shows the corresponding registration function

Considering the multi-resolution optimization strategy and the low SNR of TRUS images, a set of experiments using synthetic data are presented to demonstrate the robustness of CRMI to low sampling resolutions and its anti-noise performance. Figure 3a, b show the translational results of CRMI when the synthetic images are subsampled by factors of 4 × 4 and 2 × 2. Figure 4a, c show the images presented in Fig. 2b after the addition of 10- and 20-dB Gaussian noise, respectively. Figure 4b, d are the corresponding translational results of CRMI. It is clear that all registration functions are relatively smooth and have a large capture range around the global minimum. Although the subsampling registration functions are not as smooth as the original, especially when subsampled by the factor of 4 × 4, the optimal alignment still corresponds to the global minimum.

### Clinical prostate data

The proposed method is validated on prostate TRUS and MR images representing 12 patients, which are obtained from Shanghai Changhai Hospital. The size of the MR images is 384 × 384 pixels, and the spatial resolution is 0.4688 × 0.4688 mm^2^; the size of the TRUS images is 383 × 691 pixels, and the spatial resolution is 0.1667 × 0.1667 mm^2^. To improve registration accuracy, the TRUS images are resampled to have the same pixel size as the MR images, and a zero-padding strategy is applied to the resampled TRUS images such that the images are the same size with the MR images. The prostate MR and TRUS images are segmented using the Chan-Vese model. For the prostate images of all 12 patients, the corresponding anatomical landmarks are selected by clinical expert.

We employ the common metrics [12] such as DSC, HD, TRE and registration time to evaluate the registration performance. DSC and HD measure the global registration accuracy, and TRE describes how well the local details are aligned. A high value of DSC means that the prostate region on a moving image maps well to that on a fixed image. A low value of HD indicates good correspondence between the coordinates of two outlines. The lower the value of TRE is, the better the local registration accuracy has been achieved.

**Fig. 5:**
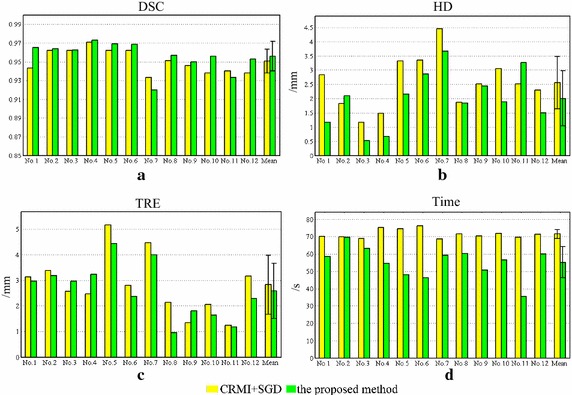
*Bar graphs* of the four evaluation criteria for minimizing CRMI using SGD and ISGD. **a** Displays the overlap rate of each sample and their mean value. **b** Shows the Hausdorff distance of each sample and their mean value. **c** Displays the registration error between landmarks of each sample and their mean value. **d** Shows the registration time of each sample and their mean value

First, we have compared the performance of minimizing CRMI between ISGD and SGD. As shown in Fig. 5, the mean registration time consumed by the proposed method is significantly shorter than SGD. This result demonstrates the effectiveness of the improvement of ISGD. The average values of DSC, HD and TRE also become better, thus verifying that ISGD has a stronger global searching ability and is more likely to jump out of local minima than SGD.

Then we compare the proposed method against two other algorithms: LMRF [11] which is widely used in 3D Slicer and CMI [13] which exhibits higher registration accuracy over MI in many cases. All three algorithms use the same multi-resolution framework to improve search efficiency, and the number of bins is set to 64.

**Fig. 6:**
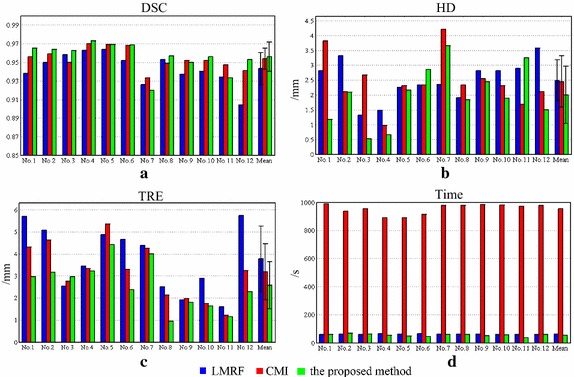
*Bar graphs* of the four evaluation criteria for the three methods. **a** Displays the overlap rate of each sample and their mean value. **b** Shows the Hausdorff distance of each sample and their mean value. **c** Displays the registration error between landmarks of each sample and their mean value. **d** Shows the registration time of each sample and their mean value

**Fig. 7:**
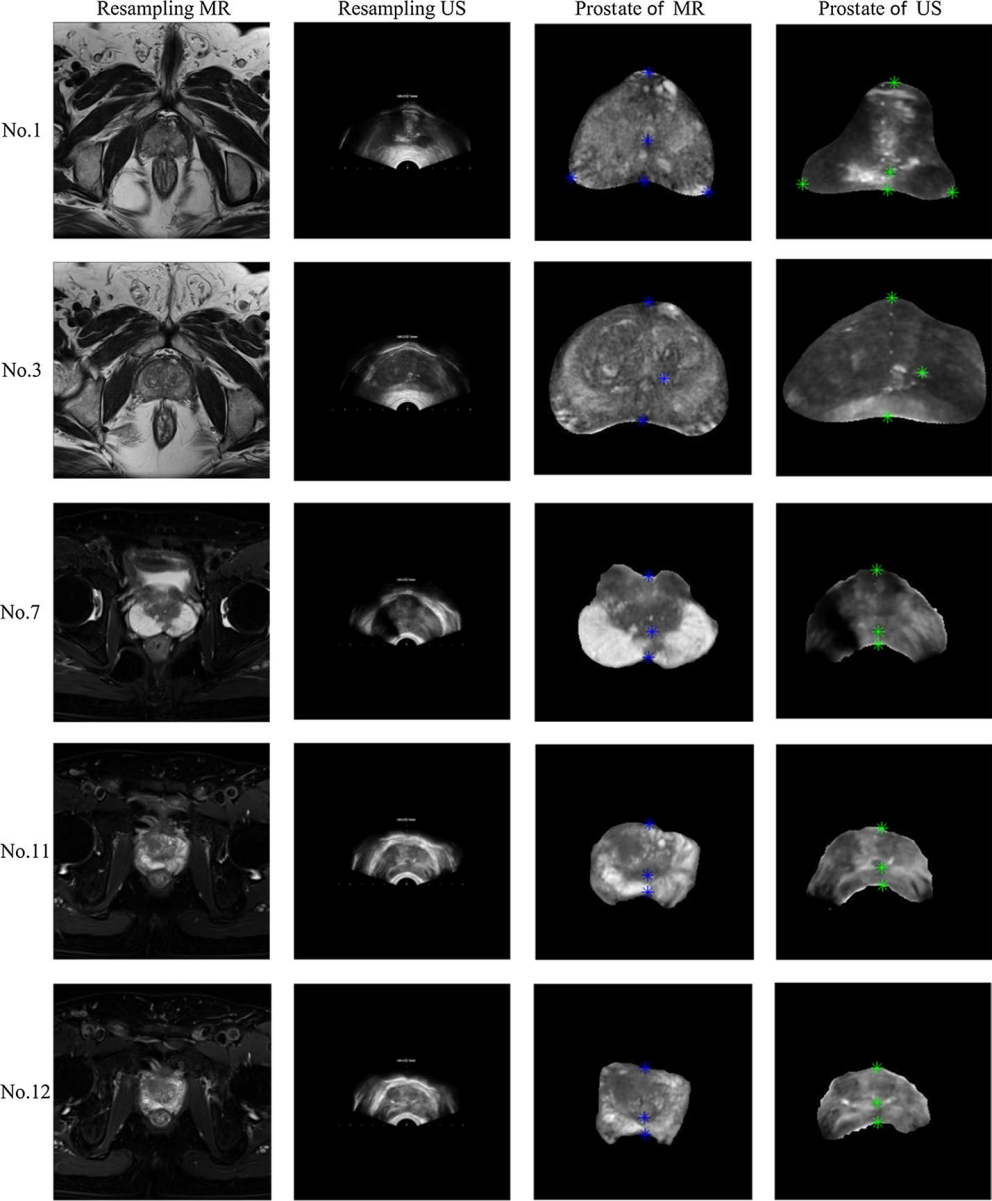
The resampling images and prostate images for the five samples. The *1st* and *2nd columns* display the resampling MR slices and TRUS slices, respectively. The *3rd* and *4th columns* show the prostate area segmented according to Chan-Vese model for both the MR and TRUS images

**Fig. 8:**
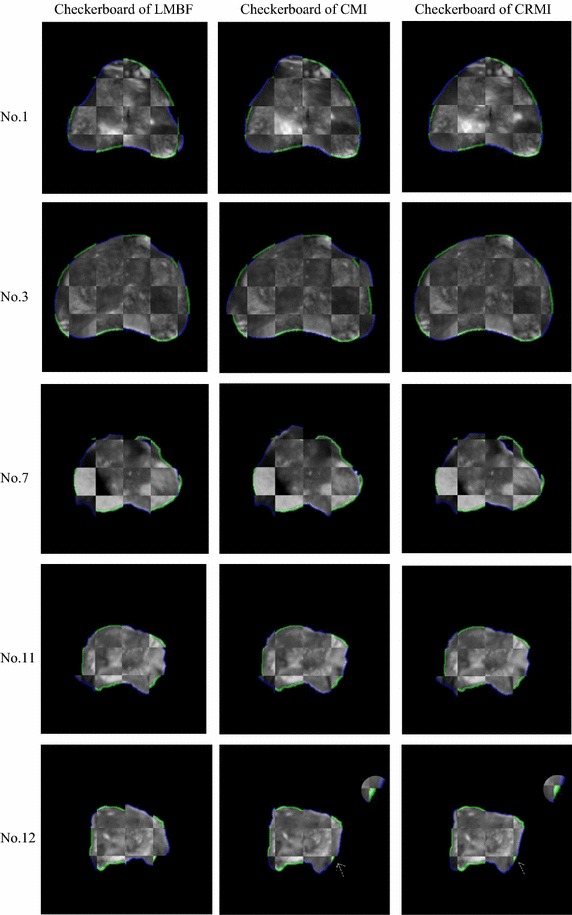
Checkerboard images of the five samples obtained using the three methods. The MR boundary is shown in green, while the TRUS is shown in *blue*. The *1st column* displays the checkerboard images obtained using LMRF. The *2nd column* displays the checkerboard images obtained using CMI. The *3rd column* displays the checkerboard images obtained using CRMI

**Fig. 9:**
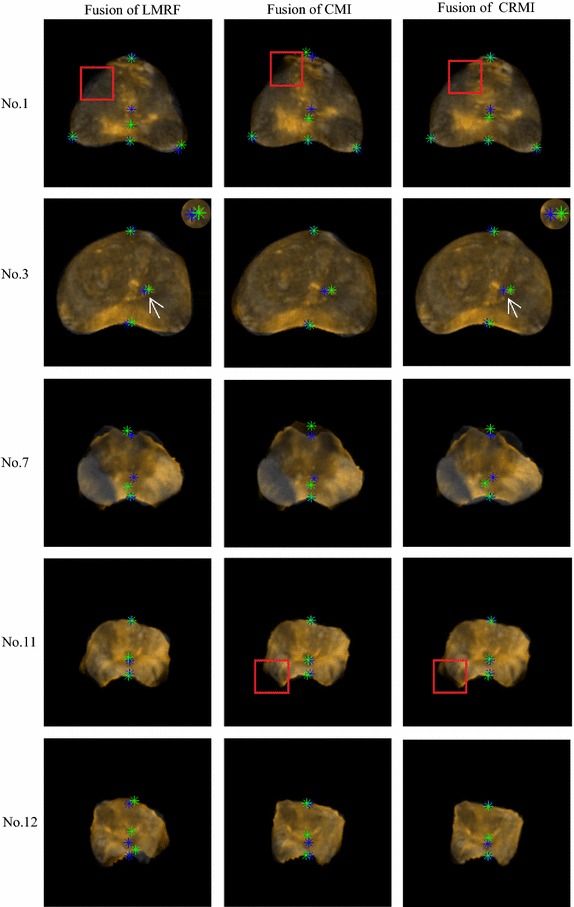
Fusion images of the five samples using the three methods. The TRUS images are enhanced with orange for observation. The *1st column* displays fusion images obtained using LMRF. The *2nd column* displays fusion images obtained using CMI. The *3rd column* displays fusion images obtained using CRMI

Figure 6 shows the evaluation criteria obtained by LMRF, CMI and the proposed method. Figure 7 presents the five groups (No. 1, No. 3, No. 7, No. 11 and No. 12) of adjusted images and the corresponding prostate images. The samples No. 1 and No. 12 are the representations to show the excellent performance of the proposed method. The samples No. 3 and No. 11 show some special cases that the proposed method does not get the best values for some metrics. And the sample No. 7 is an extreme case that the proposed method fails to correct for the deformation. Figure 8 shows the registration performance of the boundary of the three methods as checkerboard images. Figure 9 presents fusion images that fuse the MR images to the registered TRUS images obtained using the three methods.

As shown in Fig. 6, the average TRE values are 3.78 ± 1.47, 3.19 ± 1.28, and 2.58 ± 1.08 mm for LMRF, CMI and CRMI, respectively, showing that the local homologous structures that are optimized using CRMI have the highest alignment accuracy. The average HD value of CRMI is also significantly reduced. Although the average DSC value of CRMI is not significantly better than that of CMI, it is increased compared with that of LMRF. The improvement in these global measures validates that the new similarity criterion aligns the global deformation more reliably at low resolution. The registration time consumed by CRMI is approximately 16 times less than that consumed by CMI and is similar to LMRF, although CRMI requires the extra calculation of CR.

For most samples, the three metrics of DSC, HD and TRE of CRMI are the best. For sample No. 1, it is easy to observe that the correspondence between the two outlines aligned using CRMI is the optimal (see No. 1 in Fig. 8), and the distances between the landmarks are the least (see No. 1 in Fig. 9). For sample No. 12, it is clear that the registration accuracy of CRMI is higher than CMI, whereas LMRF fails to correct the displacements, thus further confirming that the new similarity criterion is better at evaluating the scatter-plot dispersion of the intensities in the presence of noise and intensity distortion.

However, some cases remain that should be considered. For sample No. 3, the global measures obtained using CRMI are the best, but the TRE value is not the least. As the partial enlarged drawing of No. 3 shown in Fig. 9, the distances between the landmarks obtained using CRMI are larger than LMRF. The reason might be that CRMI tends to search more accurate global movement parameters at low resolution because of the sample robustness; however, it falls into a local extremum at high resolution due to the inappropriate step factor *sk*. For samples No. 11, the checkerboard image obtained using CRMI is more approximate, and the lower left of the fusion image exhibits a smaller overlap rate than CMI, corresponding to the bar graphs shown for DSC and HD. It occurs because the parameter *T*_0_ decides whether ISGD receives the current solution or not is too large; therefore, the current result will be received even if the cost function changes a lot, eventually leading to premature convergence at low resolution. Unexpectedly, as the checkerboard images of sample No. 1 shown in Fig. 8, the checkerboard image obtained using CMI is smoother than LMRF, while the HD values are opposite. This occurs because the maximum distance between the outlines is not observable when the number of checkerboards is inappropriate.

The sample No. 7 is an extreme case that the values of DSC, HD and TRE obtained using CRMI are far worse than their average values. Considering that the other two algorithms also fail to align this group of images, the reason might be that the images are seriously affected by noise and intensity distortion, so that the intensities could not satisfy the assumption of intensity class correspondence or the functional mapping between aligned pixel pairs.

## Discussion and conclusion

In this study, we propose and verify a new similarity metric termed CRMI for 2D MR and TRUS prostate alignment. This method corrects the scatter-plot dispersion of the intensities caused by the deformation of location and intensity bias. Obtaining knowledge about the functional mapping between the intensities of float images and reference images is crucial when searching for the optimal alignment. We incorporate the functional dependence of the intensities quantified by CR into the intensity class correspondence held by MI. Experiments based on simulated data demonstrate that CRMI is more smooth and reliable for aligning images with intensity distortion. The new metric is also robust to noise due to its calculation of the functional correlation in *L* ^2^ space, which indicates that the intensity values of noise will be replaced by those of their neighboring samples because we map the pixel pairs with the smallest distance in intensity space. Furthermore, the computational error between the estimation of the joint density and the real density caused by insufficient sampling will be corrected during the searching of intensity mapping, which ensures that CRMI is not sensitive to down-sampling and is more suitable for multi-resolution optimization strategy.

Another innovation presented here is the improvement of the global searching ability of SGD with less computation time. We use the exact value of the cost function within a closed-loop system to select a suitable step size for each iteration as well as the stop criteria. Therefore, the improved SGD optimizer can adaptively determine the optimal number of iterations for different data, which will avoid hemstitching phenomenon due to the use of redundant iterations. An important parameter to determine the best step is the step factor *sk*. At low resolution, this value is set to 0.7 to save time. At medium and high resolution, this value is set to 0.17 to capture a more accurate step size. While searching for the minimum of the cost function, we not only accept smaller values but also consider relatively large values at a certain probability level by using Metropolis acceptance criteria. Therefore, the improved SGD optimizer is more likely to jump out of the local extremum because of the additional random disturbance. The constant *T*_0_ directly affects the global searching ability of ISGD. Considering that the exact cost function value changes within a value of 0.001, this constant is set to 0.0006, 0.00054 and 0.0008 for low, medium and high resolution, as experimentally determined. Comparative experiments using clinical prostate data verify that ISGD yields more accurate optimization performance and requires less registration time.

After comparing two non-rigid registration algorithms of LMRF and CMI, the proposed method of minimizing CRMI using ISGD provides the largest average overlap rate and the least mean HD value. The alignment accuracy of the landmarks also has a significant improvement. The average registration time is similar to that using LMRF, which is approximately 16 times less than that for CMI. These improvements confirm that the proposed method is more suitable for aligning 2D MR and TRUS images. The main limitation is that a few special cases may not fulfil the assumption of the functional mapping between aligned pixel pairs. The proposed algorithm may be extended to 3D MR and TRUS registration which can provide a wide range of information about the prostate, and the parallel computing capability of GPU can be exploited to realize real-time registration for TRUS-guided prostate biopsy.

## Appendix 1

Exploiting the independence of *λ*_**x**,*f*_ and *N*_*f*_ of *ξ*_**x**_, we first compute $\partial \mu_{f} /\partial \xi_{{\mathbf{x}}}$ as follows:

$$\frac{{\partial \mathop \mu \nolimits_{f} }}{{\partial \mathop \xi \nolimits_{{\mathbf{x}}} }} = \partial \frac{{\frac{{\sum\nolimits_{{{\mathbf{x}} \in V}} {\mathop \lambda \nolimits_{{{\mathbf{x}},f}} \mathop \xi \nolimits_{{\mathbf{x}}} } }}{{\mathop N\nolimits_{f} }}}}{{\partial \mathop \xi \nolimits_{{\mathbf{x}}} }} = \frac{{\mathop \lambda \nolimits_{{{\mathbf{x}},f}} }}{{\mathop N\nolimits_{f} }}.$$

According to Eq. (19), the key to calculating $\partial (1 - CR)/\partial \xi_{{\mathbf{x}}}$ is to calculate $\partial \sigma^{2} /\partial \xi_{{\mathbf{x}}}$ and $\partial \left( {\frac{1}{N}\sum\nolimits_{{{\mathbf{x}} \in V}} {\mathop \xi \nolimits_{{\mathbf{x}}}^{2} } - \sum\nolimits_{f} {\mathop N\nolimits_{f} \mathop \mu \nolimits_{f}^{2} } } \right)/\partial \mathop \xi \nolimits_{{\mathbf{x}}}$. Therefore, these are derived independently.

$$\frac{{\partial \mathop \sigma \nolimits^{2} }}{{\partial \mathop \xi \nolimits_{{\mathbf{x}}} }} = \partial \frac{{\frac{1}{N}\sum\nolimits_{{{\mathbf{x}} \in V}} {\mathop \xi \nolimits_{{\mathbf{x}}}^{2} } - \bar{m}^{2} }}{{\partial \mathop \xi \nolimits_{{\mathbf{x}}} }} = \frac{2}{N}\mathop \xi \nolimits_{{\mathbf{x}}} - \frac{2}{N}\bar{m}$$

$$\begin{aligned} \partial \frac{{\left( {\frac{1}{N}\sum\nolimits_{{{\mathbf{x}} \in V}} {\mathop \xi \nolimits_{{\mathbf{x}}}^{2} } - \sum\nolimits_{f} {\mathop N\nolimits_{f} \mathop \mu \nolimits_{f}^{2} } } \right)}}{{\partial \mathop \xi \nolimits_{{\mathbf{x}}} }} &= \frac{2}{N}\mathop \xi \nolimits_{{\mathbf{x}}} - \sum\nolimits_{f} {\mathop N\nolimits_{f} \cdot 2\mathop \mu \nolimits_{f} \frac{{\partial \mathop \mu \nolimits_{f} }}{{\partial \mathop \xi \nolimits_{{\mathbf{x}}} }}} \hfill \\ &= \frac{2}{N}\mathop \xi \nolimits_{{\mathbf{x}}} - \sum\limits_{f} {\mathop N\nolimits_{f} \cdot 2\mathop \mu \nolimits_{f} \frac{{\mathop \lambda \nolimits_{{{\mathbf{x}},f}} }}{{\mathop N\nolimits_{f} }}} = \frac{2}{N}\mathop \xi \nolimits_{{\mathbf{x}}} - 2\sum\nolimits_{f} {\mathop \lambda \nolimits_{{{\mathbf{x}},f}} } \mathop \mu \nolimits_{f} \hfill \\ &= \frac{2}{N}\mathop \xi \nolimits_{{\mathbf{x}}} - 2\left( {\mathop \lambda \nolimits_{{{\mathbf{x}},\mathop f\nolimits_{1} - 1}} \mathop \mu \nolimits_{{\mathop f\nolimits_{1} - 1}} + \mathop \lambda \nolimits_{{{\mathbf{x}},\mathop f\nolimits_{1} }} \mathop \mu \nolimits_{{\mathop f\nolimits_{1} }} } \right) \hfill \\ \end{aligned}$$

Therefore, $\partial (1 - CR)/\partial \xi_{{\mathbf{x}}}$ can be calculated as follows:

$$\begin{aligned} \frac{\partial (1 - CR)}{{\partial \mathop \xi \nolimits_{{\mathbf{x}}} }}\big|_{{\mathop \xi \nolimits_{{\mathbf{x}}} = \mathop I\nolimits_{m} ({\mathbf{T}}({\mathbf{x}};{\mathbf{u}}))}} &= \frac{{ - 2(\mathop \xi \nolimits_{{\mathbf{x}}} - \bar{m})}}{{\mathop {N\sigma }\nolimits^{4} }}\left( {\frac{1}{N}\sum\limits_{{{\mathbf{x}} \in V}} {\mathop \xi \nolimits_{{\mathbf{x}}}^{2} } - \sum\limits_{f} {\mathop N\nolimits_{f} \mathop \mu \nolimits_{f}^{2} } } \right) + \frac{1}{{\mathop \sigma \nolimits^{2} }}\left( {\frac{{2\mathop \xi \nolimits_{{\mathbf{x}}} }}{N} - 2\left( {\mathop \lambda \nolimits_{{{\mathbf{x}},\mathop f\nolimits_{1} - 1}} \mathop \mu \nolimits_{{\mathop f\nolimits_{1} - 1}} + \mathop \lambda \nolimits_{{{\mathbf{x}},\mathop f\nolimits_{1} }} \mathop \mu \nolimits_{{\mathop f\nolimits_{1} }} } \right)} \right) \hfill \\ &= \frac{2}{{N\mathop \sigma \nolimits^{2} }}\left( {\mathop \xi \nolimits_{{\mathbf{x}}} - N\left( {\mathop \lambda \nolimits_{{{\mathbf{x}},\mathop f\nolimits_{1} - 1}} \mathop \mu \nolimits_{{\mathop f\nolimits_{1} - 1}} + \mathop \lambda \nolimits_{{{\mathbf{x}},\mathop f\nolimits_{1} }} \mathop \mu \nolimits_{{\mathop f\nolimits_{1} }} } \right) - \frac{1}{{\mathop \sigma \nolimits^{2} }}\left( {\mathop \xi \nolimits_{{\mathbf{x}}} - \bar{m}} \right)\left( {\frac{1}{N}\sum\limits_{{{\mathbf{x}} \in V}} {\mathop \xi \nolimits_{{\mathbf{x}}}^{2} } - \sum\limits_{f} {\mathop N\nolimits_{f} \mathop \mu \nolimits_{f}^{2} } } \right)} \right). \hfill \\ \end{aligned}$$

## Appendix 2

Considering the independence of *B*_*z*_(*v*) and **u** of *x*, we calculate the first-order derivative of the deformation field with respect to *x* using the chain rule as follows:

$$\begin{aligned} \frac{{\partial {\mathbf{T}}}}{\partial x} &= \sum\limits_{l = 0}^{3} {\sum\limits_{z = 0}^{3} {\frac{{\partial \mathop B\nolimits_{l} (w)}}{\partial w}\frac{\partial w}{\partial x}\mathop B\nolimits_{z} (v)} } \mathop u\nolimits_{{\mathop p\nolimits_{x} + l,\mathop p\nolimits_{y} + z}} \\ &= \sum\limits_{l = 0}^{3} {\sum\limits_{z = 0}^{3} {\frac{{\partial \mathop B\nolimits_{l} (w)}}{\partial w}\frac{\partial }{\partial x}\left( {\frac{x}{\delta } - \left\lfloor {x/\delta } \right\rfloor } \right)\mathop B\nolimits_{z} (v)} } \mathop u\nolimits_{{\mathop p\nolimits_{x} + l,\mathop p\nolimits_{y} + z}} \\ & = \frac{1}{\delta }\sum\limits_{l = 0}^{3} {\sum\limits_{z = 0}^{3} {\frac{{\partial \mathop B\nolimits_{l} (w)}}{\partial w}\mathop B\nolimits_{z} (v)\mathop u\nolimits_{{\mathop p\nolimits_{x} + l,\mathop p\nolimits_{y} + z}} } } .\end{aligned}$$

The derivative with respect to *y* can be computed in the same way:

$$\frac{{\partial {\mathbf{T}}}}{\partial y} = \sum\limits_{l = 0}^{3} {\sum\limits_{z = 0}^{3} {\mathop B\nolimits_{l} (w)\frac{{\partial \mathop B\nolimits_{z} (v)}}{\partial v}\frac{\partial v}{\partial y}\mathop u\nolimits_{{\mathop p\nolimits_{x} + l,\mathop p\nolimits_{y} + z}} } } = \frac{1}{\delta }\sum\limits_{l = 0}^{3} {\sum\limits_{z = 0}^{3} {\mathop B\nolimits_{l} (w)\frac{{\partial \mathop B\nolimits_{z} (v)}}{\partial v}\mathop u\nolimits_{{\mathop p\nolimits_{x} + l,\mathop p\nolimits_{y} + z}} } } .$$

Then, we can gain the second-order derivative

$$\begin{aligned} \frac{{\mathop \partial \nolimits^{2} {\mathbf{T}}}}{{\partial \mathop x\nolimits^{2} }} &= \frac{1}{\delta }\sum\limits_{l = 0}^{3} {\sum\limits_{z = 0}^{3} {\frac{{\mathop \partial \nolimits^{2} \mathop B\nolimits_{l} (w)}}{{\partial \mathop w\nolimits^{2} }}\frac{\partial w}{\partial x}\mathop B\nolimits_{z} (v)} } \mathop u\nolimits_{{\mathop p\nolimits_{x} + l,\mathop p\nolimits_{y} + z}} \\ & = \frac{1}{{\mathop \delta \nolimits^{2} }}\sum\limits_{l = 0}^{3} {\sum\limits_{z = 0}^{3} {\frac{{\mathop \partial \nolimits^{2} \mathop B\nolimits_{l} (w)}}{{\partial \mathop w\nolimits^{2} }}\mathop B\nolimits_{z} (v)} } \mathop u\nolimits_{{\mathop p\nolimits_{x} + l,\mathop p\nolimits_{y} + z}} \end{aligned}$$

$$\begin{aligned} \frac{{\mathop \partial \nolimits^{2} {\mathbf{T}}}}{{\partial \mathop y\nolimits^{2} }} &= \frac{1}{\delta }\sum\limits_{l = 0}^{3} {\sum\limits_{z = 0}^{3} {\mathop B\nolimits_{l} (w)\frac{{\mathop \partial \nolimits^{2} \mathop B\nolimits_{z} (v)}}{{\partial \mathop v\nolimits^{2} }}\frac{\partial v}{\partial y}\mathop u\nolimits_{{\mathop p\nolimits_{x} + l,\mathop p\nolimits_{y} + z}} } } \\ &= \frac{1}{{\mathop \delta \nolimits^{2} }}\sum\limits_{l = 0}^{3} {\sum\limits_{z = 0}^{3} {\mathop B\nolimits_{l} (w)\frac{{\mathop \partial \nolimits^{2} \mathop B\nolimits_{z} (v)}}{{\partial \mathop v\nolimits^{2} }}\mathop u\nolimits_{{\mathop p\nolimits_{x} + l,\mathop p\nolimits_{y} + z}} } } \end{aligned}$$

$$\begin{aligned} \frac{{\mathop \partial \nolimits^{2} {\mathbf{T}}}}{\partial x\partial y} &= \frac{1}{\delta }\sum\limits_{l = 0}^{3} {\sum\limits_{z = 0}^{3} {\frac{{\partial \mathop B\nolimits_{l} (w)}}{\partial w}\frac{{\partial \mathop B\nolimits_{z} (v)}}{\partial v}\frac{\partial v}{\partial y}\mathop u\nolimits_{{\mathop p\nolimits_{x} + l,\mathop p\nolimits_{y} + z}} } } \\ & = \frac{1}{{\mathop \delta \nolimits^{2} }}\sum\limits_{l = 0}^{3} {\sum\limits_{z = 0}^{3} {\frac{{\partial \mathop B\nolimits_{l} (w)}}{\partial w}\frac{{\partial \mathop B\nolimits_{z} (v)}}{\partial v}\mathop u\nolimits_{{\mathop p\nolimits_{x} + l,\mathop p\nolimits_{y} + z}} } } \end{aligned}.$$

An arbitrary control point *u*_*i*,*j*_ influences the pixels in a local neighbourhood, which is defined as $\left\{ {{\mathbf{x}} \in \varOmega |\,\left| {{\mathbf{x}} - u_{i,j} } \right| \le 2\delta } \right\}$. Therefore, the derivative of the above second-order derivative with respect to *u*_*i*,*j*_ is

$$\frac{{\partial \left( {\partial ^{2} {\mathbf{T}}/\partial x^{2} } \right)}}{{\partial {\text{ }}u_{{i,j}} }} = \left\{ {\begin{array}{*{20}l} {\frac{1}{{\delta ^{2} }}\frac{{\partial ^{2} {\text{ }}B_{{i - {\text{ }}p_{x} }} (w)}}{{\partial {\text{ }}w^{2} }}{\text{ }}B_{{j - {\text{ }}p_{y} }} (v),} \quad & {\left| {{\mathbf{x}} - {\text{ }}u_{{i,j}} } \right| \le 2\delta } \\ {0,} & \quad {otherwize} \\ \end{array} } \right.$$

$$\frac{{\partial \left( {\partial ^{2} {\mathbf{T}}/\partial y^{2} } \right)}}{{\partial {\text{ }}u_{{i,j}} }} = \left\{ {\begin{array}{*{20}l} {\frac{1}{{\delta ^{2} }}{\text{ }}B_{{i - {\text{ }}p_{x} }} (w)\frac{{\partial ^{2} {\text{ }}B_{{j - {\text{ }}p_{y} }} (v)}}{{\partial {\text{ }}v^{2} }},{\mkern 1mu} } & \quad {\left| {{\mathbf{x}} - {\text{ }}u_{{i,j}} } \right| \le 2\delta } \\ {0,} & \quad {otherwize} \\ \end{array} } \right.$$

$$\frac{{\partial \left( {\partial ^{2} {\mathbf{T}}/\partial x\partial y} \right)}}{{\partial {\text{ }}u_{{i,j}} }} = \left\{ {\begin{array}{*{20}l} {\frac{1}{{\delta ^{2} }}\frac{{\partial {\text{ }}B_{{i - {\text{ }}p_{x} }} (w)}}{{\partial w}}\frac{{\partial {\text{ }}B_{{j - {\text{ }}p_{y} }} (v)}}{{\partial v}},} & \quad {{\mkern 1mu} \left| {{\mathbf{x}} - {\text{ }}u_{{i,j}} } \right| \le 2\delta } \\ {0,} & \quad {otherwize} \\ \end{array} } \right..$$

## Abbreviations

TRUS: transrectal ultrasound
PSA: prostate specific antigen
MR: magnetic resonance
MI: mutual information
LMRF: label map registration frame
ICP: iterative closest point
TRE: target registration error
NMI: normalized mutual information
DSC: dice similarity coefficient
L-BFGS: Limited-Broyden-Fletcher-Goldfarb-Shanno
CMI: conditional mutual information
SNR: signal to noise ratio
CRMI: correlation ratio-based mutual information
SGD: stochastic gradient descent
CR: correlation ratio
ASGD: adaptive stochastic gradient descent
ISGD: improved stochastic gradient descent
HD: Hausdorff distance
